# Striatal Acetylcholine and Dopamine Interactions Produce Situation-appropriate Action Selection

**DOI:** 10.2174/1570159X21666230912093041

**Published:** 2023-09-13

**Authors:** Laura A. Bradfield, Serena Becchi, Michael D. Kendig

**Affiliations:** 1 School of Life Sciences, Faculty of Science, University of Technology Sydney, New South Wales 2007, Australia;; 2 Decision Neuroscience Laboratory, School of Psychology, University of New South Wales Sydney, Sydney, NSW 2052, Australia

**Keywords:** Action-outcome contingency, acetylcholine, dopamine, striatum, thalamus, action-outcome associations

## Abstract

Individuals often learn how to perform new actions for particular outcomes against a complex background of existing action-outcome associations. As such, this new knowledge can interfere or even compete with existing knowledge, such that individuals must use internal and external cues to determine which action is appropriate to the current situation. The question thus remains as to how this problem is solved at a neural level. Research over the last decade or so has begun to determine how the brain achieves situation-appropriate action selection. Several converging lines of evidence suggest that it is achieved through the complex interactions of acetylcholine and dopamine within the striatum in a manner that relies on glutamatergic inputs from the cortex and thalamus. Here we briefly review this evidence, then relate it to several very recent findings to provide new, speculative insights regarding the precise nature of striatal acetylcholine/dopamine interaction dynamics and their relation to situation-appropriate action selection.

## 1 INTRODUCTION

**Fig. (1):**
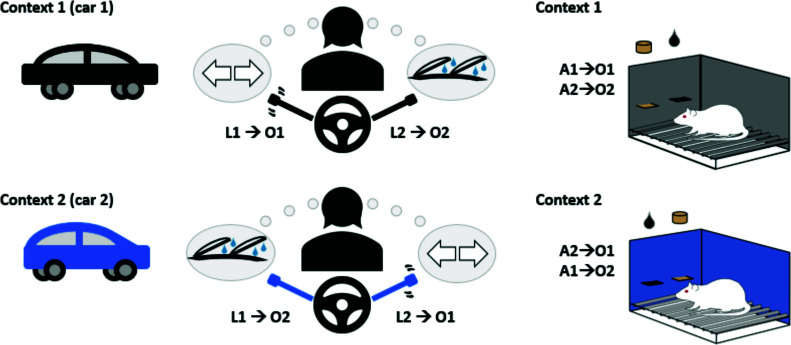
Contextual modulation of action selection in humans (left) and rats (right). Left Panel: Someone who normally pulls on the left-hand stalk to indicate and on the right-hand stalk to turn on the windscreen wipers may encounter the reverse configuration when borrowing a friend’s car. This is but one of many ways in which action selection can be context-specific. Right Panel: This form of contextual learning can be modelled in rats by training two instrumental action-outcome associations in different contexts; in context 1 response A1 produces outcome 1 and response A2 produces outcome 2, but in context 2 these contingencies are reversed.

An individual passing the office vending machine on their way out of work might choose to buy a chocolate bar on Monday, a muesli bar on Tuesday, and a packet of chips on Wednesday. Snack in hand, that same individual arrives at their car and drives home, pushing up the stalk to the left of the steering wheel to activate the indicator. On Thursday, however, they take their partner’s car to work, in which they need to push up the stalk to the right of the steering wheel to indicate. They also bring their laptop to work, careful to make sure they enter the laptop password and not the password from their desktop computer. Through these and many similar examples, it is clear that we hold multiple, competing associations between actions and outcomes in our minds simultaneously, and it is only through the modulation of those associations by internal and external contextual cues that we produce the action that earns the most appropriate outcome to the current situation (Fig. **1**, Left Panel). Although this function has been the subject of recent reviews [1, 2], how exactly the contextual modulation of action-outcome associations is achieved at a neural level is still being determined. Here we provide an update to the opinions expressed in these recent reviews in light of several recent new findings (shown in Table **1**) regarding cholinergic and dopaminergic interactions within the striatum. Please refer to the methodology section for the criteria used to select publications for the current review.

## 2 POSTERIOR DORSOMEDIAL STRIATUM AS THE HUB OF SPECIFIC ACTION-OUTCOME CONTINGENCY KNOWLEDGE

What is clear from several decades of research is that the posterior dorsomedial striatum (pDMS) comprises a neuroanatomical hub of action-outcome contingency knowledge, as it is here that such knowledge is both formed and stored. The initial evidence for this was observed in experimentally naïve rats that received permanent or temporary inactivation of their pDMS and was then taught to press left and right levers for food outcomes (sucrose, pellets, and/or fruit punch) that were novel to the animal [3, 4]. After several days of lever press training, rats were fed to satiety on one of these outcomes to reduce its value [5] and then given a choice test in which both levers were extended, but responses did not earn any outcomes. Control animals with an intact pDMS were able to selectively respond on the lever that had been associated with the still-valued outcome during training, and to avoid the lever associated with the devalued outcome, suggesting that they a) were sensitive to the current value of the outcome and b) could recall the action-outcome contingency from earlier training. As these are the two criteria of goal-directed action [5], these animals were said to be acting in a goal-directed manner. By contrast, pDMS-inactivated animals responded equally on both levers, suggesting that their capacity for goal-directed action was impaired.

These findings showed that the pDMS is necessary for the learning of salient and novel action-outcome contingencies. However, learning and decision-making in the real world are rarely so straightforward. Rather, most learning incorporates new information into a rich tapestry of prior learning about associations between events, actions, and outcomes. These relationships are rarely fixed or uniform. Rather, we regularly learn new associations that interfere or compete with those already learned. As demonstrated by our above-mentioned examples, it is not adaptive for this new learning to simply overwrite prior learning because different situations might demand different actions to achieve the same outcome, such as pushing up a left or right stalk in different cars to turn on an indicator, or because the same actions might be associated with different outcomes, such as inserting money into a vending machine for chips *versus* chocolate. The recognition of this problem, therefore, provided a new challenge for behavioural scientists: to not only determine how the brain learns per se but also how it learns to juggle multiple, competing contingencies and to apply them appropriately to each situation.

Recent work has suggested that the answer to this question also resides in the striatum, specifically its microcircuits, which are in turn controlled externally through inputs from the thalamus, cortex, and midbrain. In particular, a number of recent studies have suggested that situation-appropriate action selection is achieved through the dynamic interactions of striatal acetylcholine (ACh) and dopamine (DA). Although the evidence for this hypothesis has already been reviewed [1, 2, 6], even in the short time, there have been many new preprints and publications that shed new light on the nature of these interactions. Here we integrate these new findings with previous work, to determine what they might reveal about how the brain achieves the situation-appropriate selection of actions based on action-outcome contingencies.

Balleine *et al.*, [1] offered a particularly elegant description of how the pDMS might accurately juggle between competing action-outcome contingencies, and it is this account that we will integrate with new findings. Specifically, they suggested that cholinergic interneurons (CINs) in the pDMS influence dopamine release through nicotinic receptors on DA terminals to modulate D2-spiny projecting neurons (SPNs). These D2-SPNs then modulate the excitation or inhibition of D1-SPNs to select the action-outcome contingency most appropriate to the current situation. We will now briefly recap the findings that led to this account.

In 2013, we [7] modified the outcome devaluation procedure outlined above in a way that allowed us to parse the neural mechanisms underlying the initial acquisition of action-outcome contingencies from those underlying the acquisition of new, competing action-outcome contingencies, as shown in Fig. (**1**) (Right Panel). Specifically, if rats initially learned to press a left lever for pellets and a right lever for sucrose (for example) we then reversed these contingencies such that the left lever now earned sucrose and the right lever earned pellets. Subsequently, a second outcome devaluation test was administered in the same manner as before, and found that although animals with dysfunctional pDMS CINs (as a result of inactivating their parafascicular thalamic [PF] inputs) exhibited goal-directed control upon testing prior to reversal, after reversal their goal-directed actions were impaired. These findings suggest that, when functional, pDMS ACh uses contextual information (*e.g*. the initial learning context or the reversal context) to determine which action-outcome contingencies are currently appropriate. This also seems to apply in humans, because studies using proton magnetic resonance spectroscopy (1 H-MRS) during a probabilistic reversal learning task strongly suggest that, as in rats and mice, fluctuating ACh levels in the dorsal striatum is critically linked to the ability to flexibly use competing contingency knowledge [8, 9].

Moreover, recent studies have shown that whereas the initial learning of action-outcome contingencies depends on D1-expressing SPNs in the pDMS, the contextual modulation of these contingencies depends on D2-expressing SPNs in the same region. Using the same devaluation/reversal paradigm described, Peak *et al.* [10] showed that chemogenetically inhibiting direct pathway-projecting SPNs in the pDMS, which predominantly express the D1 receptor, impaired the initial acquisition of action-outcome contingencies. By contrast, inhibiting the indirect pathway projecting SPNs in pDMS that predominantly express the D2 receptor did not affect initial devaluation performance, but led to a loss of sensitivity after reversal. Matamales *et al.*, [11] confirmed this latter result directly, observing impaired sensitivity to devaluation after the reversal in adora2a-Cre::drd2-eGFP mice given bilateral lesions of D2-SPNs in the pDMS *via* injections of Casp3-TEVp virus. They further demonstrated that D2 SPNs produce this function by modulating the activity of specific ensembles of D1-SPNs. Balleine *et al.* interpreted these findings as evidence that subpopulations of D1 SPNs might contain the instantiation of memory for specific action-outcome contingencies, much in the same way particular neuronal ensembles (or their synapses) within the hippocampus instantiate specific context-fear memories as part of an ‘engram’ [12]. They further concluded that D2-SPN modulation of these “action-outcome contingency engrams”, which is itself modulated by CIN activity, provides the situationally-appropriate contextual information to ensure the correct action-outcome contingency is executed.

## 3 RECENT FINDINGS REGARDING HOW POSTERIOR DORSOMEDIAL STRIATUM PRODUCES SITUATION-SPECIFIC ACTION SELECTION

Recent preprints and publications add to these findings and provide exciting new insights in this space. One key indication they have made is how exactly the firing dynamics of ACh and DA might interact to produce this contextual modulation. For instance, it has long been speculated that the characteristic ‘burst-pause’ firing pattern observed in striatal CINs provides a window that allows DA to enhance (or possibly inhibit [13]) plasticity at cortico-striatal and thalamo-striatal synapses [14]. Recently, Liu *et al.* [15] showed that CINs appear to do this directly, by depolarising DA axons in the striatum rather than relying on somatic release of DA from cell bodies in the midbrain. In relation to the contextual modulation of goal-directed actions, this finding suggests that when new or competing action-outcome contingencies are being learned, striatal CINs could perhaps broadcast dopamine in a manner that might enable plasticity in specific populations of D1 SPNs. In support of this notion, Becchi *et al.* [16] recently discovered that simply reversing the identities of outcomes earned by actions in rats is sufficient to elicit burst-pause firing in CINs, and that lesioning or inflaming parafascicular inputs to CINs impairs acquisition of a goal directed action when it changes.

In further support, and from the same paper, the infusion of monoamine oxidase (MAO) B inhibitor selegiline, which increases the levels of DA in the brain, rescued both the irregularity in burst-pause firing patterns as well as the behavioural impairment. Unfortunately, this conclusion seems to be complicated by several findings. For example, Becchi *et al.* [16] also demonstrated that selegiline’s ability to rescue contextual modulation of goal-directed action was unlikely to be mediated by DA because the *in vitro* application of D1 antagonist, SCH23390, or D2 antagonist, raclopride, onto striatal slices did not prevent selegiline-induced burst-pause activity of CINs. It was, however, abolished by the application of ouabain, a Na^+^/K^+^ ATPase inhibitor, suggesting that selegiline was instead acting through a different mechanism. These exciting findings indicate the potential for new therapeutic opportunities, although with the caveat that caution should be exercised when *in vitro* findings are used to infer information about *in vivo* firing dynamics and their relation to behaviour. In particular, the ability of selegiline to rescue goal-directed flexibility through increasing Na^+^/K^+^ ATPase pump activity suggests that Selegiline could be administered at different stages of Parkinson’s disease to resist cognitive inflexibility [17], or even during normal ageing as decision-making becomes less flexible [18] and where there is evidence of decreased Na^+^/K^+^ ATP pump function [19].

Recently, a different study that used *in vivo* recordings also raises complications for Balleine *et al.* [5] working account. Chantranupong *et al.*, [20], showed that in the ventral striatum, the interactions between DA and ACh are bidirectional, raising the possibility of DA modulating CIN activity rather than the other way around. To elucidate the directionality of DA/ACh modulation during the situation-appropriate selection of actions, future studies could utilise *in vivo* recording techniques similar to those employed by Chantranupong *et al.*, [20] in the dorsomedial rather than ventral striatum in conjunction with a suitable behavioural paradigm such as the outcome-reversal task described by Bradfield *et al.*, [7], Matamales *et al.*, [11], and Peak *et al.*, [10]. If DA signalling preceded ACh signalling (or *vice versa*) prior to each lever press, and if this directionality was specific to post-reversal testing, this would reveal whether DA modulation of ACh or ACh modulation of DA underpinned the situation-appropriate selection of each action.

A third recent finding that complicates this account was reported by Krok *et al.* [21], who identified phasic changes in striatal DA and ACh which were coherent even in the absence of movement and salient stimuli. Surprisingly, this coherence was maintained across behavioural contexts, a finding that is potentially problematic for the notion that DA/ACh interactions provide contextual information to guide action selection because, if that were the case, one would expect these interactions to change across contexts. One important caveat, however, is that the recordings of Krok *et al.*, were made in the dorsolateral striatum, and in the same paper the authors report that these interactions do not necessarily occur in the dorsomedial striatum in the same way.

Despite these complexities, one relatively clear finding that did arise from these new publications is that striatal ACh/DA interactions appear to be different in their modulation of learning driven by model-free reward-prediction errors compared to that driven by model-based state-prediction errors. In particular, Chantranupong *et al.*, [20] found that DA transients that reflected reward prediction error (RPE) signalling (*i.e*. a pattern of firing to unpredicted rewards, not firing to predicted reward, and firing to stimuli that reliably predicted reward) were unaffected by the broad striatal loss of ACh. The authors suggested that DA RPE signalling is likely to emerge from DA soma in the midbrain, rather than be elicited from ACh modulation of DA axon terminals. By contrast, ACh-dependent DA function mediated by the D2 receptor did impair the ability of the animals to modify switching behaviour, presumably through an RPE-independent mechanism. Interestingly, the study by Matamales *et al.*, [11] also found that the RPE-driven behaviour led to a distinct and more intermingled transcription profile of D1 and D2-SPNs compared to the more regionally-specific profile driven by state prediction error, and it is state prediction error that we [7, 22] have previously suggested underlying the learning of situation-specific action selection.

Although here we have provided an update to the model proposed by Balleine *et al.*, [1] which focussed on the modulation of DA/ACh interactions *via* nicotinic receptors, it is important to acknowledge the contribution of muscarinic receptors to flexible action selection. Indeed, there is considerable evidence implicating striatal muscarinic receptors in cognitive flexibility, *e.g*. [23, 24], and this role appears to differ functionally from that played by nicotinic receptors [25]. Of particular relevance to the current review is a study by Mamaligas *et al.*, [26] who report that individual CINs within the striatum make long-distance muscarinic synapses with multiple, overlapping patches of spiny projecting neurons (SPN)s, particularly direct pathway SPNs *via* the inhibitory Gi-coupled M4 receptor (also here building on earlier anatomical work of Matamales *et al.*, [27]). They further discovered that the strength of these connections varies from CIN to CIN, so that even weak CIN firing can result in significant inhibitory modulation of multiple SPNs. If those SPNs carry “action-outcome contingency engrams” that compete with each other, as proposed, then this could be the mechanism by which irrelevant or inappropriate action-outcome contingencies are inhibited. To put it simply, if a rat has learned that both a right and a left lever earn sucrose, but currently only presses on the left lever that is earning sucrose, then the rat must inhibit the “right lever-sucrose” memory in order to press the left lever. This inhibition could thus be achieved through ACh release from CINs leading to M4-mediated inhibition of the direct pathway SPN ensemble that has stored the “right-lever-sucrose” memory.

## CONCLUSION

In summary it is clear that as the sophistication of our tools and techniques evolve, so too does our understanding of the neural mechanisms that underlie processes such as the contextual modulation of action selection. New findings are providing novel insights into the *in vivo* interactions of neuromodulators DA and ACh within the striatum, while also raising questions to be addressed by future studies. One obvious question is how regionally specific patterns of these interactions are within the striatum, a question that ideally could be answered within the same study using the same techniques, allowing for direct comparisons. Another similar question is how, and to what extent, these interactions are driven by external inputs into the striatum – not only from the midbrain DA neurons but also by glutamatergic inputs from the cortex and thalamus (note that Chantranupong *et al.*, have already begun to answer this question, [20]). However, it is worth noting that the mechanisms underlying situation-appropriate action selection are likely not limited solely to those discussed here, particularly given that nicotinic receptors are expressed by other subtypes of striatal interneurons that also play distinctive roles in action selection [28, 29]. Lastly, we would like to note that the combination of these techniques with highly controlled and sophisticated behavioural paradigms (such as the reversal of action-outcome contingency learning followed by devaluation) is necessary to reveal exactly what these regionally specific, externally driven, interactions between DA and ACh mean in terms of the cognitive-behavioural outputs.

## METHODOLOGY OF THE REVIEW

Publications were selected for the current review (shown in Table **1**) according to the following criteria: 1) published within the last 3 years (*i.e*. from 2020 onwards), 2) they reveal novel findings about cholinergic and/or dopaminergic function within the striatum, and 3) they reveal novel information – either directly or indirectly – about the interactions between dopamine and acetylcholine within the striatum, with a focus on the dorsal striatum.

## Figures and Tables

**Table 1:** A summary of the key findings from the articles selected for the current review, as well as their interpretation within a posterior dorsomedial striatal account of situation-appropriate action selection.

**References**	**Key Findings**	**Interpretation of These Findings Within a Striatal Account of Selecting Situation-appropriate Actions**
Becchi *et al.*, 2022.	Reversing action-outcome contingencies induce burst firing in dorsomedial striatal cholinergic interneurons (CINs) in a parafascicular thalamic nucleus input-dependent manner. Intra-striatal infusions of the monoamine oxidase (MAO) B inhibitor selegiline rescues impairments in both behaviour and in the burst-firing of CINs in a dopamine-independent mechanism.	Situation-appropriate action selection relies on the burst-firing of dorsomedial striatal CINs. Such a firing pattern could allow for plasticity in specific ensembles of spiny projection neurons which then encode the currently appropriate action-outcome contingencies in a manner that is not dependent on dopamine.
Chantranupong *et al.*, 2022	In the ventral striatum, certain characteristics of dopamine firing do not rely on the release of acetylcholine by cholinergic interneurons (CINs). However, dopamine does inhibit acetylcholine levels through D2 receptors on CINs.	The raises the possibility that contextual modulation of action selection could occur through dopaminergic modulation of CINs (rather than the other way around, as proposed by Balleine *et al.*, 2021).
Krok *et al.*, 2022	Coherent, phasic changes in striatal dopamine and acetylcholine were observed in the dorsolateral striatum in a manner that was not dependent on movement or salient stimuli, and was maintained across multiple behavioural contexts.	These findings are potentially problematic for the notion that dopamine/acetylcholine dynamics reflect the contextual modulation of action selection because if so, these dynamics should fluctuate across behavioural contexts. However, this is noted with the caveat that such dynamics may differ from those in the dorsomedial striatum, where the current account is focused.
Liu *et al.*, 2022	Cholinergic interneurons in the striatum depolarise dopamine axons within the striatum rather than relying on the somatic release of dopamine from cell bodies in the midbrain.	Striatal CINs could broadcast dopamine when competing action-outcome contingencies are being learned. This could allow for plasticity in specific D1 SPNs – plasticity which could underlie the ‘engram’ for action-outcome contingencies.
Matamales *et al.*, 2020	Lesioning D2, indirect pathway SPNs impairs goal-directed action after contingency reversal but not before. Also, D2 SPNs directly modulate the activity of specific ensembles of D1-SPNs.	Provides a mechanism by which D2 SPNs can modulate D1 SPNs directly in order to choose the ensemble containing the action-outcome contingency memory (engram) that is most appropriate to the current situation.
Peak *et al.*, 2020	Direct pathway-projecting SPNs in the dorsomedial striatum (that predominantly express the D1 receptor) encode specific action-outcome contingency memories. By contrast, indirect pathway-projecting SPNs in the same region (that predominantly express the D2 receptor) appear to modulate the contextual selection of specific action-outcome contingencies.	Suggests that direct pathway, D1 SPNs contain the instantiation of specific action-outcome contingencies whereas indirect pathway, D2 SPNs provide the necessary contextual information for the situation-appropriate selection of these contingencies.

## LIST OF ABBREVIATIONS

ACh: Acetylcholine
CINs: Cholinergic Interneurons
DA: Dopamine
MAO: Monoamine Oxidase
pDMS: Posterior Dorsomedial Striatum
PF: Parafascicular Thalamic
RPE: Reward Prediction Error
SPNs: Spiny Projecting Neurons
